# The 27-gene IO score is associated with efficacy of PD-1/L1 inhibitors independent of FGFR expression in a real-world metastatic urothelial carcinoma cohort

**DOI:** 10.1007/s00262-023-03401-x

**Published:** 2023-02-19

**Authors:** Tyler J. Nielsen, Matthew G. Varga, Catherine T. Cronister, Brian Z. Ring, Robert S. Seitz, Douglas T. Ross, Brock L. Schweitzer, Kimberly McGregor

**Affiliations:** Oncocyte Corporation, 15 Cushing, Irvine, CA 92618 USA

**Keywords:** IO score, DetermaIO, *FGFR3*, Metastatic urothelial carcinoma mUC, PD-1/PD-L1 inhibitors, Immunotherapy

## Abstract

Multiple targeted therapeutics have been approved by the FDA for mUC, including immune checkpoint inhibitors (ICIs) and more recently targeted agents for both FGFR and Nectin-4. *FGFR3*-aberrant and Nectin-4 expressing cells have been associated with an immunosuppressed phenotype. Given that less than half of all patients respond to these agents as monotherapies and less than 20% are eligible to receive salvage therapy, effective personalized treatment plans are critical. Typical biomarkers for ICIs such as PD-L1 and TMB have not been definitive in mUC, yet a biomarker-driven optimization of first-line therapy and subsequent sequencing have the potential to achieve higher and more durable response rates. The IO score is a 27-gene tumor immune microenvironment (TIME) classifier that has been associated with the clinical benefits of ICIs in multiple cancer types, including mUC. This study demonstrates that the IO score was associated with both progression-free survival (PFS) and overall survival (OS) in a real-world cohort of mUC patients treated with ICIs. Furthermore, the IO score was independent of and provided information incremental to TMB. Interestingly, the IO score predicted benefit in patients with high FGFR expression, despite conflicting data regarding response rates among the FGFR aberrant population. Taken together, these results demonstrate that the IO score assessment of the TIME is associated with a clinical benefit from ICI therapy and that this novel biomarker may inform therapeutic sequencing decisions in mUC, potentially improving outcomes for this notoriously difficult-to-treat disease.

**Supplementary Information:**

The online version contains supplementary material available at 10.1007/s00262-023-03401-x.

## Introduction

Current clinical guidelines for the treatment of metastatic urothelial carcinoma (mUC) recommend first-line treatment with a platinum-containing regimen followed by immune checkpoint inhibitor (ICI) maintenance therapy (avelumab) for patients who achieve a response, whereas an ICI (atezolizumab or pembrolizumab) is recommended for platinum-ineligible patients [1–4]. Although ICI therapy is also recommended for patients who fail to achieve a first-line response to platinum therapy, clinical study results have been mixed in the second-line setting. For example, the FDA application of atezolizumab in the second-line setting was withdrawn due to failure to achieve an improvement in overall survival (OS) in the IMvigor211 clinical study [5]. However, alternative targeted options are now available, including the FGFR inhibitor (FGFRi) erdafitinib, for patients who have progressed on platinum with susceptible *FGFR3* or *FGFR2* genetic alterations and the monoclonal antibody–drug conjugate, enfortumab vedotin (EV) targeting Nectin-4, is approved for patients progressing after chemotherapy and immunotherapy or for those who are cisplatin ineligible and have been treated with one or more prior therapies [6, 7].

Although the response rates to these new targeted agents are promising, evidence-based algorithms for managing therapeutic options are lacking, and the strategy for optimized sequencing of cytotoxic, targeted, and immune therapy options has the potential to be informed by improved response biomarkers. Response rates of 44% to EV have been observed in Nectin-4 positive patients in second-line or higher settings [8]. In the 20% of patients with FGFR alterations who were either platinum ineligible or failed prior to platinum therapy, erdafitinib showed a 40% response rate [9]. In *FGFR3* altered patients, the tumor immune microenvironment (TIME) has been found to have a generally non-T cell inflamed phenotype, but also relatively low immunosuppressive stromal features, which may allow a subset of patients to be responsive to ICI therapy [10–14]. Optimizing the use of these therapeutic options might benefit from improved biomarkers to assess the likelihood of response to immune therapy and composite biomarker analyses to better optimize therapeutic sequencing, inform rational combinations, and increase the response to the initial therapeutic treatment.

The 27-gene immuno-oncology classifier (IO score) quantifies gene expression to classify the TIME and uses an algorithm that combines results into a continuous score with a pre-specified binary IO positive (IO +) or IO negative (IO− ) classification to predict response to immune therapy [15]. The IO score classification has demonstrated an association with ICI efficacy in multiple tumor types including non-small cell lung cancer (NSCLC), triple negative breast cancer (TNBC), and urothelial carcinoma (UC) [15–21]. Using data from a real-world UNC-108 cohort, the results confirmed the findings of the IMvigor210 study that the IO score is associated with the efficacy of ICI therapy in mUC [18]. Furthermore, this was accomplished using the same binary classification threshold used in all previous studies, and this study will discuss the potential of an evidence-based likelihood of response assessment to inform therapeutic sequencing in the treatment of advanced mUC [15–21].

## Methods

### Patient dataset

**Fig. 1:**
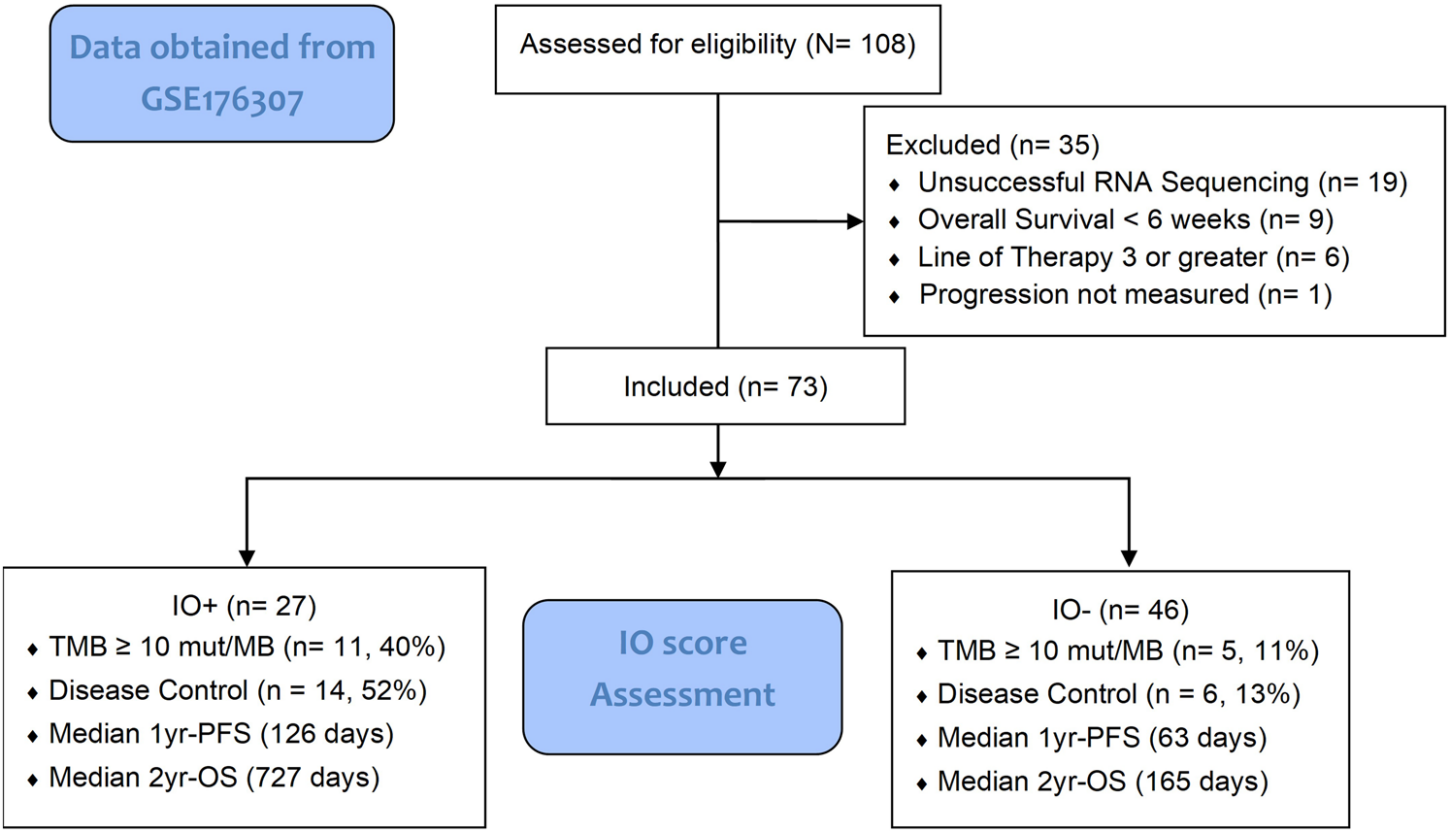
CONSORT 2010 flow diagram for analysis of IO score from UNC-108 cohort

The UNC-108 dataset was downloaded from the Gene Expression Omnibus (GEO) (RRID:SCR_005012) under accession ID GSE176307. Patient inclusion and exclusion criteria have been defined previously [14]. Briefly, the UNC-108 dataset is composed of 108 advanced mUC patients who received single-agent ICI therapy (anti-PD1 or anti-PDL-1) between January 2014 and June 2018 and for whom response data were available. Of the 108 patients, 89 had tumor tissues available for whole-transcriptome sequencing. This real-world cohort did not have publicly available PD-L1 immunohistochemistry staining data. Patients who survived less than six weeks or were treated beyond the second-line setting were prospectively excluded to reduce the confounding effects of insufficient follow-up or multiple pre-treatment agents for the assessment of IO score or tumor mutational burden (TMB) on disease progression, as described in Fig. 1 (CONSORT, RRID:SCR_018720) [22].

### IO score classification

The IO score is a 27-gene immuno-oncology assay intended to predict response to immune checkpoint inhibitors as described previously [15]. The IO score classifies cases as positive or negative based on their correlation to established gene expression centroids for immunomodulatory, mesenchymal, and mesenchymal stem-like subtypes [23]. The classification threshold was previously established in breast and lung cancers and validated as a classifier for mUC using TCGA and IMvigor210 datasets [15–19].

To assess data for the UNC-108 cohort, RNA expression analysis was used to determine the IO score of patients with available data downloaded from GSE176307 using the requisite 27-genes as previously described (ITM2A gene missing, Cohen’s *κ* = 0.99 from TCGA-BLCA (RRID:SCR_003193; Project: Bladder Urothelial Carcinoma, Table S1). Differential gene expression was assessed using the DESeq (RRID:SCR_000154) package and were z-score normalized. The IO score uses a proprietary algorithm to classify cases as positive or negative based on their similarity to established centroids for immunomodulatory, mesenchymal, and mesenchymal stem-like gene expression centroids [23]. A model considering patients who were positive by IO score and/or TMB high was also classified as IO + /TMB^high^ when patients had reported TMB scores greater than 10 mut/MB.

### TCGA–BLCA analysis

Gene expression was obtained from TCGA-BLCA (*n* = 433) by FPKM. Each sample was classified by IO score as IO positive (*n* = 176) or IO negative (*n* = 257) using the previously established threshold. The FPKM for *NECTIN4*, *FGFR3*, and *FGFR1* was obtained and grouped by IO score positivity. Differences in IO positivity were tested using the Wilcoxon rank-sum method for each gene.

### Statistical analysis

Patient demographics and clinical characteristics were compared using the chi-square test. R version 4.2.0 (2022-04-02) with packages survival (RRID:SCR_021137) and survminer (RRID:SCR_021094) and R-Studio software (2022.02.3) were used for survival analyses. To evaluate the efficacy of ICI therapy, overall survival (OS) and progression-free survival (PFS) were calculated using Cox proportional hazards modeling with 95% confidence intervals (95% CIs). A bivariate model was used to combine TMB and IO scores to test for independence. Survival data were plotted using the Kaplan–Meier estimator method. RECIST v1.1 criteria were used to define objective response and subsequently calculate the disease control rate (complete response (CR), partial response (PR), or stable disease (SD) versus progressive disease (PD)).

## Results

### Patient summary

A cohort of patients with advanced urothelial carcinoma (UC) were identified within the University of North Carolina Hospital system as having received treatment with at least one dose of monotherapy PD-(L)1 ICI (UNC-108 cohort) between January 2014 and June 2018. Of the 73 qualified patients (Fig. 1), 27 (36.9%) were positive for the IO score, 16 (22%) reported as TMB high (≥ 10 mut/Mb), and 32 were positive for IO + and/or TMB high (Table 1). Clinical parameters and demographic data were similar between IO score positive and negative, except for RECIST-based response and TMB high, which were both more likely to be IO positive (*p* = 0.0047 and *p* = 0.0029, respectively).

**Table 1:** Demographics of UNC-108 cohort qualifying for analysis. Defined in terms of the entire cohort or by IO score

	Cohort (*n* = 73)	IO + (*n* = 27)	IO− (*n* = 46)	*p*-value^*^
*Age at IO therapy (years)*				*p* = 0.88
≤ 50	3 (4%)	1 (4%)	2 (4%)	
51–60	12 (16%)	5 (19%)	7 (15%)	
61–70	24 (33%)	10 (37%)	14 (30%)	
> 70	34 (47%)	11 (41%)	23 (50%)	
*Sex*				*p* = 0.41
Female	28 (38%)	12 (44%)	16 (35%)	
Male	45 (62%)	15 (56%)	30 (65%)	
*Race*				*p* = 0.53
African American	18 (25%)	5 (19%)	13 (28%)	
Caucasian	51 (70%)	21 (78%)	30 (65%)	
Other	4 (5%)	1 (4%)	3 (7%)	
*Disease stage at Dx*				*p* = 0.70
Stage 0	1 (1%)	0	1 (2%)	
Stage 1	5 (7%)	3 (11%)	2 (4%)	
Stage 2	47 (64%)	19 (70%)	28 (61%)	
Stage 3	15 (21%)	4 (15%)	11 (24%)	
Stage 4	4 (5%)	1 (4%)	3 (7%)	
Unavailable	1 (1%)	0	1 (2%)	
*Histology*				*p* = 0.38
Transitional cell carcinoma (TCC)	51 (70%)	20 (74%)	31 (67%)	
TCC with squamous	10 (14%)	5 (19%)	5 (115)	
TCC with other	11 (15%)	2 (7%)	9 (20%)	
Non-TCC	1 (1%)	0	1 (2%)	
*Primary tumor location*				*p* = 0.62
Bladder	63 (86%)	24 (89%)	39 (85%)	
Ureter/renal pelvis	10 (14%)	3 (11%)	7 (15%)	
*FGFR3 status*				*p* = 0.61
Aberrant	14 (19%)	6 (22%)	8 (17%)	
Wild type	59 (81%)	21 (78%)	38 (83%)	
*ECOG performance status*				*p* = 0.23
0	18 (25%)	5 (19%)	13 (28%)	
1	28 (38%)	9 (33%)	19 (41%)	
2	11 (15%)	4 (15%)	7 (15%)	
3	2 (3%)	2 (7%)	0	
Unavailable	14 (19%)	7 (26%)	7 (15%)	
*ICI therapy received*				*p* = 0.15
Atezolizumab	26 (36%)	9 (33%)	17 (37%)	
Durvalumab	2 (3%)	0	2 (4%)	
Nivolumab	5 (7%)	0	5 (11%)	
Pembrolizumab	40 (55%)	18 (67%)	22 (48%)	
*ICI line of therapy*				*p* = 0.63
1	18 (25%)	8 (30%)	10 (22%)	
2	55 (75%)	19 (70%)	36 (78%)	
*Response status*				*p* = 0.0047
CR	7 (10%)	5 (19%)	2 (4%)	
PR	9 (12%)	6 (22%)	3 (7%)	
SD	4 (5%)	3 (11%)	1 (2%)	
PD	53 (73%)	13 (48%)	40 (87%)	
*TMB (high* ≥ *10 mut/MB)*				*p* = 0.0029
TMB high	16 (22%)	11 (41%)	5 (11%)	
TMB low	57 (78%)	16 (59%)	41 (89%)	

^*^Chi-square *p*-value per characteristic between IO + and IO−

To determine if any of the clinical characteristics from this cohort were significantly associated with 2-year OS or 1-year PFS, Cox proportional hazards were applied (Figure S1), which showed that only the ICI line of therapy (LoT) was significantly associated with 1-year PFS. Bivariate analysis with either the IO score or TMB combined with ICI LoT identified a loss of significance for LoT with TMB high TMB at 1-year PFS (ICI LoT HR = 2.00; 95%CI 1.06–3.79; *p* = 0.033; IO score HR = 0.41; 95%CI 0.23–0.73; *p* = 0.0023; for TMB high, ICI LoT HR = 1.71; 95%CI 0.90–3.23; *p* = 0.099; TMB high HR = 0.26; 95%CI 0.12–0.56; *p* = 0.00058). Notably, most patients were treated with atezolizumab (*n* = 26, 36%) or pembrolizumab (*n* = 40, 55%). A bivariate equation was performed with the two therapies, and the IO score remained independent of the choice of therapy for 1-year PFS (IO HR = 0.44, 95%CI 0.25–0.78, *p* = 0.0052; choice of ICI HR = 1.78, 95%CI 0.99–3.19, *p* = 0.054) and 2-years OS (IO HR = 0.38, 95%CI 0.19–0.76, *p* = 0.0059; choice of ICI HR = 1.57, 95%CI 0.80–3.10, *p* = 0.19).

### Assessment of response to therapy

Disease control rate (DCR) is the percentage of patients who achieved a complete response (CR), partial response (PR), or stable disease (SD) following ICI therapy. Given the high rate of progressive disease (PD) observed in this cohort as indicated in Table 1, DCR was the only response variable powered at > 80% and was therefore chosen as the measure for response. The disease control rates for the IO score, TMB high, and the composite IO + and/or TMB high (IO + /TMB^high^) groups were 52%, 69%, and 53%, respectively (Table 2). Interestingly, the negative predictive value (NPV) for patients who were IO−  and TMB low was 93%, which indicated that this model would be ideal for initial screening to identify likely non-responders to ICI therapy.

**Table 2:** Objective response characteristics for IO score, TMB, and IO + /TMB^high^

Biomarker	IO score	TMB high	IO + /TMB^high^
DCR for biomarker ( +) (PPV)	52% (14/27)	69% (11/16)	53% (17/32)
DCR for biomarker (-)	13% (6/46)	16% (9/57)	7% (3/41)
Odds ratio for DCR	7.18 (95%CI 2.3–22.5; *p* = 0.0007)	11.73 (95%CI 3.3–42.0; *p* = 0.0002)	14.36 (95%CI 3.7–56.2; *p* = 0.0001)
NPV	87% (40/46)	84% (48/57)	93% (38/41)

### Outcome metrics with TMB high and IO scores

#### 2-year overall survival

**Fig. 2:**
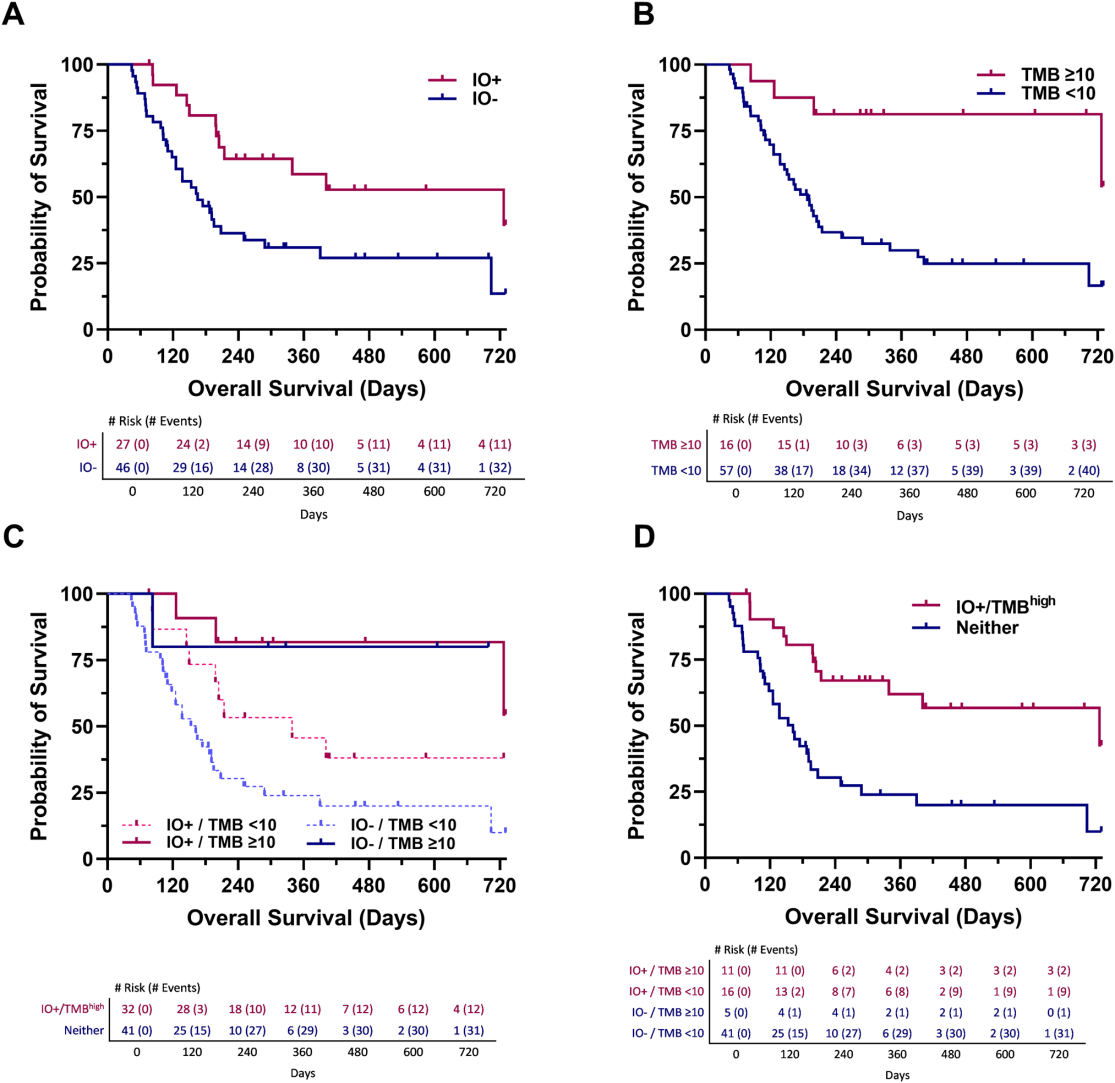
Kaplan–Meier graphs estimating 2-year overall survival for **A** IO score, **B** TMB high (≥ 10 mut/mb), **C** Bivariate IO score and TMB high, and **D** IO + and/or TMB high model

To determine whether the IO score and/or TMB were associated with survival within the UNC-108 cohort, we assessed the IO score and TMB using Cox proportional hazards for 2-year OS and 1-year PFS. When assessing 2-year OS, both IO score and TMB high were significantly associated with survival by Cox proportional hazards (IO score HR = 0.40, 95%CI 0.20–0.78, *p* = 0.005; TMB high HR = 0.22, 95%CI 0.077–0.61, *p* = 0.002; Fig. 2A, B). The median 2-year OS for IO−  patients was 165 days, whereas IO + patients was 727 days. When the IO score and TMB high were analyzed in a bivariate model, only TMB remained significant with 2-year OS (IO score HR = 0.52, 95%CI 0.26–1.036, *p* = 0.06; TMB high HR = 0.27, 95%CI 0.094–0.78, *p* = 0.02) (Fig. 2C). Through assessment of the Kaplan–Meier estimation, it was observed that patients who were IO−  and TMB low had the worst outcomes. Therefore, a model was created in which patients were stratified based on a negative IO score and TMB low (IO− /TMB^low^) or positive IO score and/or TMB high (IO + /TMB^high^). The Kaplan–Meier plot in Fig. 2D demonstrates strong separation, and the IO + /TMB^high^ model was significantly associated with 2-year OS (HR = 0.31, 95%CI 0.16–0.60, *p* = 0.0005). To further investigate the independence of the IO + /TMB^high^ model between IO score and TMB high, two bivariate Cox proportional hazards were applied and showed that only the IO + /TMB^high^ model retains its association with 2-yr OS when analyzed with TMB high (TMB high HR = 0.36, 95%CI 0.11–1.19, *p* = 0.09; IO + /TMB^high^ HR = 0.47, 95%CI 0.22–1.0, *p* = 0.05; IO score HR = 2.16, 95%CI 0.28–16.71, *p* = 0.46; IO + /TMB^high^ HR = 0.16, 95%CI 0.021–1.15, *p* = 0.07). It is worth noting that out of the 73 patients, only 5 were IO−  and TMB high (6.8%) in our cohort.

#### 1-year progression-free survival

**Fig. 3:**
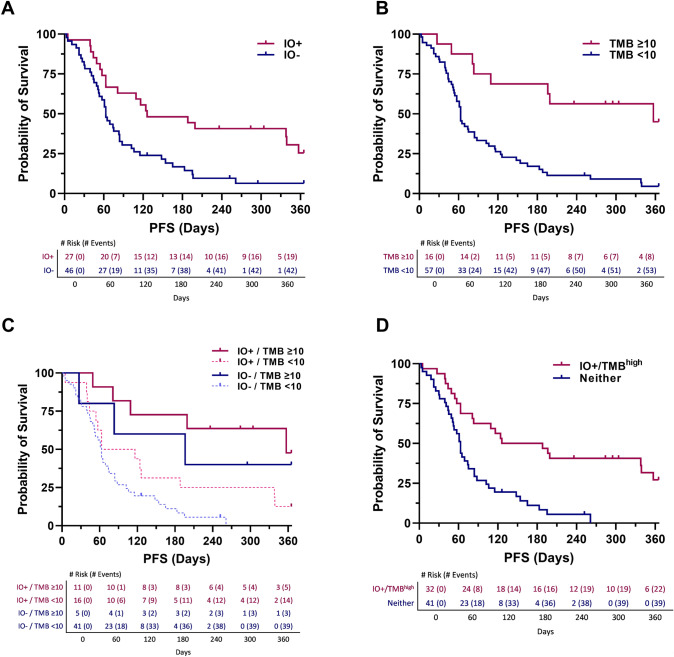
Kaplan–Meier graphs estimating 1-year progression-free survival for **A** IO score, **B** TMB high (≥ 10 mut/mb), **C** bivariate IO score and TMB high, and **D** IO + /TMB^high^ model

When considering IO score and TMB high for 1-year PFS, both biomarkers were significantly associated with progression-free survival (IO score HR = 0.41, 95%CI 0.24–0.74, *p* = 0.002; TMB high HR = 0.25, 95%CI 0.11–0.53, *p* = 0.0001) (Fig. 3A, B). Combining the biomarkers IO score and TMB high into a single bivariate equation to measure independence, both variables remained significantly associated with 1-yr PFS (IO score HR = 0.53, 95%CI 0.30–0.96, *p* = 0.04; TMB high HR = 0.29, 95%CI 0.13–0.63, *p* = 0.002) (Fig. 3C). The IO + /TMB^high^ model was also applied and was significantly associated with 1-year PFS (HR = 0.32, 95%CI 0.18–0.58, *p* = 0.0001) (Fig. 3D). In order to further investigate the independence of the factors within the IO + /TMB^high^ model, a bivariate Cox proportional hazard equation was applied to IO score (IO score HR = 1.33, 95%CI 0.44–4.02, *p* = 0.62; IO + /TMB^high^ HR = 0.27, 95%CI 0.11–0.66, *p* = 0.004) and TMB^high^ (TMB high HR = 0.36, 95%CI 0.15–0.86, *p* = 0.02; IO + /TMB^high^ HR = 0.52, 95%CI 0.28–1.0, *p* = 0.05), finding only IO score loses significance. Taken together, these data indicate that the IO score is independent and incremental to TMB high and that the IO score is the major contributor to the IO + /TMB^high^ model, likely due to a greater number of positive patients identified by the IO score.

### Assessment of IO score in FGFR expression high or aberrant patients

There was a total of 14 (19%) patients with a FGFR mutation or fusion from the UNC-108 cohort who qualified for our analysis (Table 1). The IO score was assessed for the ability to discern the benefit in FGFR-mutated patients who received ICI. Although only trending toward significance, likely due to the low number of patients in this subgroup analysis, these data suggest that there may be a clinically meaningful benefit from the IO score or the IO + /TMB^high^ model (IO HR 0.3001, 95%CI 0.0773–1.165, *p* = 0.0819; IO + /TMB^high^ HR 0.3432, 95%CI 0.09792–1.203, *p* = 0.0946) (Figure S2A). While the IO score and IO + /TMB^high^ model approached significance, TMB high alone did not in this subgroup (TMB HR 0.6162, 95%CI 0.1985–1.913, *p* = 0.402) (Figure S2B).

**Fig. 4:**
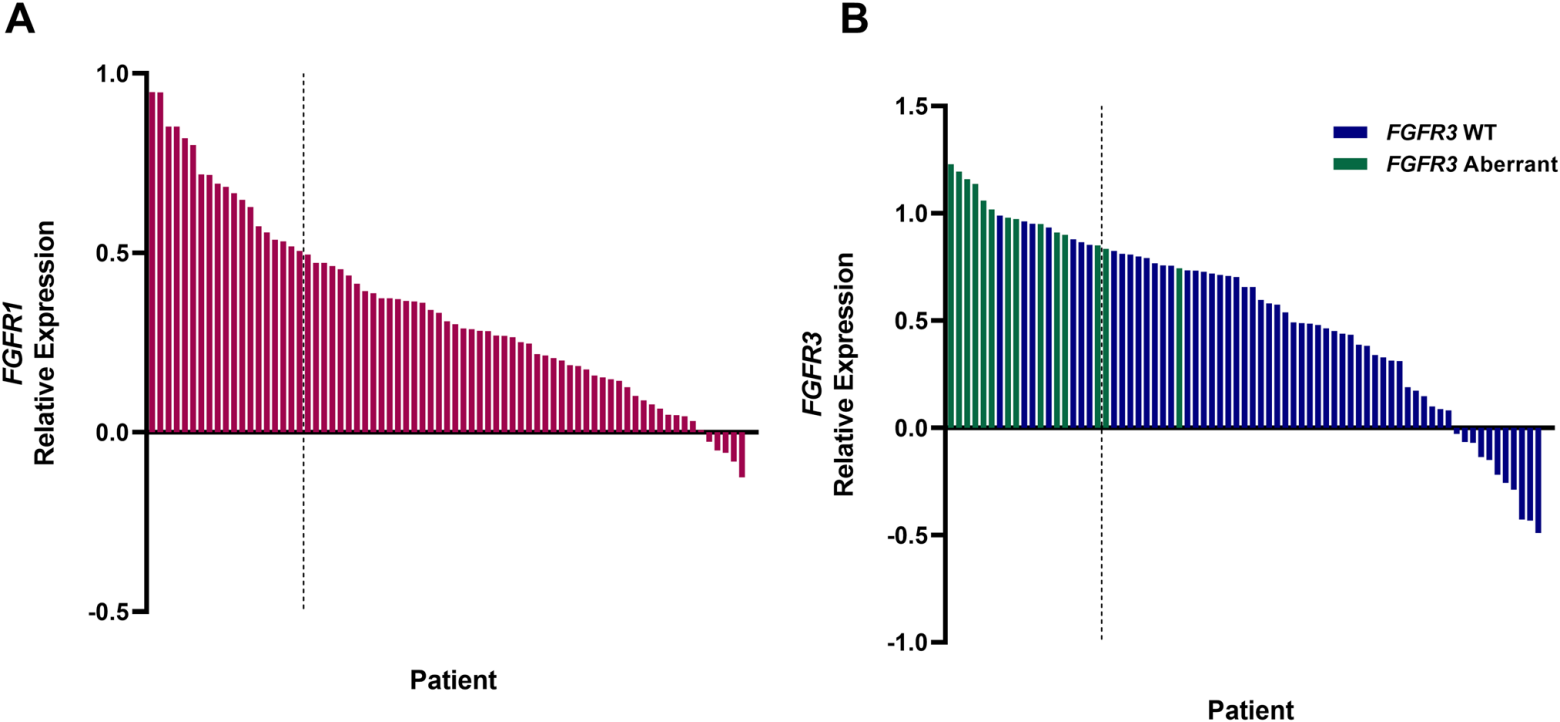
Patients with FGFR overexpression were defined by the upper quartile for **A** FGFR1 and **B** FGFR3 expression; patients with FGFR3 mutations are shown as green bars

*FGFR3* mutations are often associated with high *FGFR3* expression [14, 24]]. In fact, the ongoing FORT-2 trial (NTC03473756) stratifies patients according to the expression levels of *FGFR1* and *FGFR3* [25]. Additionally, high levels of *FGFR1/3* expression were correlated with the IO score classification by TCGA-BLCA analysis (*FGFR1/3* lower quartile IO score = 0.089, upper quartile IO score = − 0.002; *p* = 0.0076). Therefore, we evaluated the UNC-108 cohort for *FGFR1* or *FGFR3* expression and whether it could be used as a practical companion to the gene expression-based IO score. To parallel the FORT-2 trial, we determined the levels of FGFR expression and defined patients in the upper quartile of as high expression for *FGFR1* or *FGFR3* (Fig. 4A, B). High FGFR expression alone was not significantly associated with 1-year PFS (HR 1.255, 95%CI 0.7546 to 2.086, p = 0.382) in the presence of ICI therapy in the UNC-108 cohort.

**Fig. 5:**
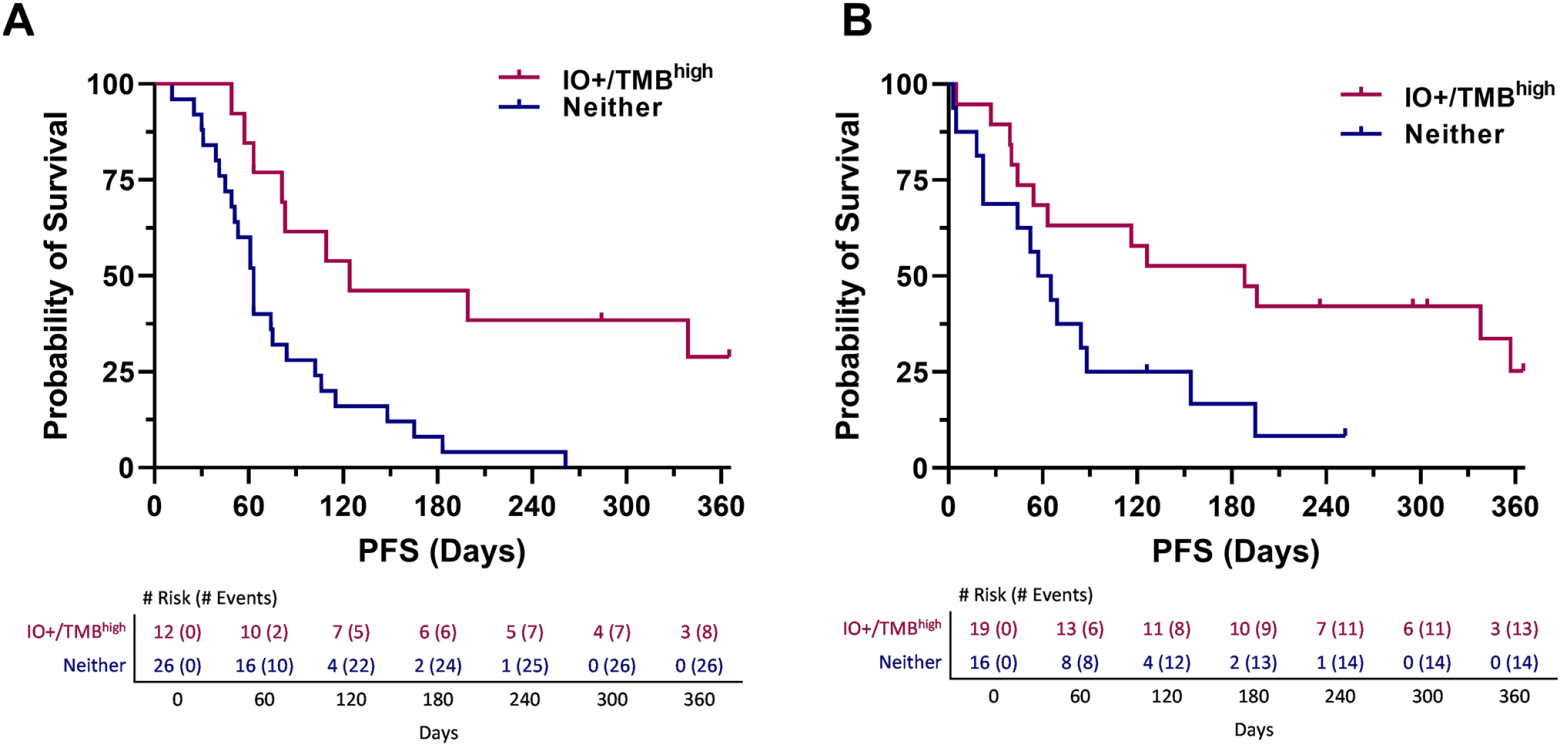
K-M estimation of IO + /TMB^high^ among patients who are **A** FGFR expression high or **B** FGFR expression normal

Among patients with high FGFR expression, the IO score and IO + /TMB^high^ biomarkers were significantly associated with 1-year PFS (*n* = 38; IO HR 0.2572, 95%CI 0.1074–0.616, *p* = 0.00231; TMB HR 0.4795, 95%CI 0.1846–1.245, *p* = 0.131; IO + /TMB^high^ HR 0.2831, 95%CI 0.1237–0.6478, *p* = 0.00281). Among the patients with normal FGFR expression, TMB high and IO + /TMB^high^ biomarkers remained significantly associated with 1-yr PFS (*n* = 35; IO HR 0.6499, 95%CI 0.2975–1.42, *p* = 0.28; TMB HR 0.08913, 95%CI 0.0201–0.3952, *p* = 0.00146; IO + /TMB^high^ HR 0.4089, 95%CI 0.1813–0.922, *p* = 0.0311). The Kaplan–Meier plot of the IO + /TMB^high^ model demonstrated an improved median 1-year PFS among patients for both the high FGFR expression (63 days to 124 days, Fig. 5A) and normal FGFR expression (61 days to 188 days, Fig. 5B).

### Assessment of NECTIN4

In addition to ICI and FGFR inhibitor therapies, enfortumab vedotin (EV) is a therapeutic option that targets Nectin-4 and has been approved for use in mUC [6]. Thus, we sought to investigate whether patients who were IO score negative had elevated *NECTIN4* expression by assessing the TCGA-BLCA dataset for IO score and *NECTIN4* expression. The IO score was calculated for each sample in the TCGA-BLCA dataset with 176 (40.6%) patients being IO + and 257 (59.4%) patients being IO− . We then assessed *NECTIN4* expression in this group by calculating the upper and lower expression quartiles. The average IO score for those patients in the upper quartile was − 0.09 (*n* = 94) and the average IO score for patients in the lower quartile was 0.15 (*n* = 103). This difference was significant according to the Mann–Whitney U test (*p* < 0.001) and was separated along the threshold for positivity of 0.09. These data indicate that a higher *NECTIN4* expression level is generally observed in the IO−  cohort, which is associated with a cold TIME [15].

## Discussion

The recent approvals of multiple targeted therapies for mUC have significantly altered therapeutic strategies; however, only a fraction of cisplatin-ineligible patients with mUC survive to reach a 2nd line of therapy [14, 26]. Therefore, there is an unmet need to improve therapeutic sequencing. Aberrant FGFR signaling has emerged as a potential biomarker for the use of FGFR inhibitors; however, biomarkers for ICIs and EV remain elusive [27–29]. Mounting evidence suggests that nectins interact with immune modulatory receptors, including TIGIT, and therefore higher levels of expression among certain tumor types may be explained by their role in immune regulation [30–32].

**Fig. 6:**
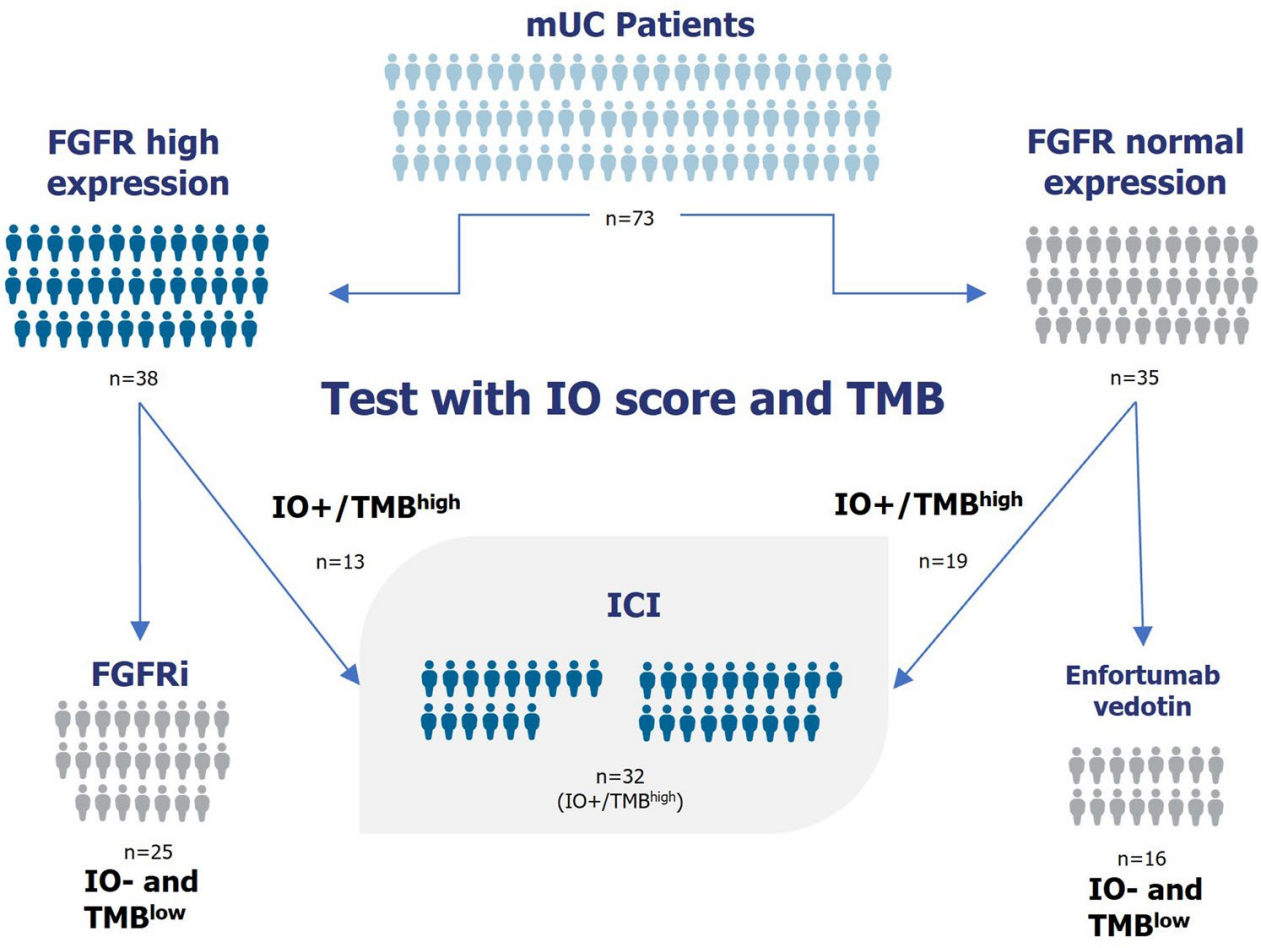
Proposed flowchart for management of mUC among platinum-ineligible patients

The 27-gene IO score has previously been shown to be associated with the efficacy of ICI therapy in multiple tumor types through its assessment of the TIME [15–18, 20, 21]. Thus, this study aimed to investigate whether the IO score could also be used either alone or as part of a composite biomarker for response to ICI therapy in mUC. By combining IO + and TMB high to assess the benefit of ICI therapy, identifying high FGFR expression, and the association of *NECTIN4* expression with the IO negative classification, we have proposed a novel biomarker schema to guide patients into evidence-based, biomarker-directed therapies (Fig. 6).

Within the UNC-108 cohort of qualified patients, both the IO score and TMB were capable of stratifying benefit from ICI therapy; however, the IO score identified a substantially larger number of patients likely to benefit compared to TMB. In total, 27 (37%) patients in the cohort were identified as IO + and 16 (22%) were identified as TMB high. There was substantial overlap between these markers, and identifying those that were IO + and/or TMB high, identified 32 (44%) positive patients (16/32 = only IO + , 5/32 = only TMB high, 11/32 = IO + and TMB high), yet TMB and IO score remained independent from each other (Fig. 3D). This confirms similar synergy between IO and TMB first identified in the IMVigor210 study [18].

Whether an mUC patient with an FGFR aberration should be prioritized for ICI therapy or FGFR inhibitors remains an unanswered question. There are conflicting reports suggesting that the TIME of *FGFR3* aberrant tumors could lead to reduced ICI therapy response rates, but the presence of T-cells inferred by mRNA expression signatures appears to be independent of *FGFR3* mutations [14, 33]. Our analysis of TCGA data demonstrated that increased *FGFR3* expression is significantly associated with the IO score negative classification, thereby suggesting a cold tumor microenvironment [15]. However, we observed a meaningful clinical benefit in the subset of high FGFR expressers who were IO + /TMB^high^ (Fig. 5A). These data, combined with a strong association between *FGFR3* expression and *FGFR3* aberrations (Fig. 4B), suggest that there is a benefit to classifying the state of the TIME within patients with aberrant FGFR to optimize the choice of ICI therapy versus FGFR targeted therapy. Interestingly, we found 34% (13 of 38) of the FGFR high expressing cases were predicted to be responders to ICI therapy.

Multiple studies in bladder cancer have indicated that patients with an Eastern Cooperative Oncology Group (ECOG) performance status (PS) of 2 have worse outcomes with ICI therapy than those with ECOG PS < 2 [34–36]. Prospective clinical trials with ECOG PS ≥ 2 are lacking, despite ICI therapy being an enticing option for the poor PS population. The UNC-108 cohort is a real-world dataset containing 11 patients with an ECOG PS of 2. Of these 11 patients, two are TMB high and four are IO positive. Despite the small cohort size, the IO score identifies meaningful benefit in this group (HR = 0.32, 95%CI 0.06–1.55, *p* = 0.16; median PFS IO−  = 52 days; IO +  = 196.5 days) (Figure S3). These are the first data we are aware of, which may help identify ICI therapy benefit specifically in the ECOG PS 2 subpopulation in mUC and are worthy of further investigation.

In an effort to describe the functional categories associated with the 27 genes of the IO score, the Gene Ontology (GO) terms for each were collected [37]. Several biological processes were common among these genes including the immune response, neutrophil chemotaxis, and the chemokine-mediated signaling pathway which are associated with the presence and activity of immune cells in the tumor microenvironment (Tables S2 and S3). Further, genes associated with extracellular exosomes, implicated in cancer progression and metastasis, align with the concept of cross-talk within the microenvironment as tumor-produced exosomes have been shown to modulate the immune response which may contribute to immune evasion [38, 39]. These functional categories support our clinical findings which suggest the IO score is a classifier that captures the interplay within the TIME and may be independent of the tumor of origin as observed in previous studies [15–19].

The present study expands on the findings of IO score assessment in the IMvigor210 trial and validates the hypothesis that the IO score alone can be an effective biomarker to identify the likely benefit of ICI therapy in patients with mUC [18]. Furthermore, an improved biomarker paradigm incorporating the IO score and TMB as a composite biomarker could serve as an effective screen with a negative predictive value (NPV) of 92.7%. A large proportion of patients who are unlikely to respond to ICIs will have high FGFR expression or FGFR aberrations, suggesting that FGFR inhibition in these patients is likely to be more beneficial. Considering the 92.7% NPV combined with an approximately 40% response rate from FGFRi and EV studies [8, 9], the proposed management schema presented here may better inform therapeutic sequencing to impact patient care and improve response rates in mUC.

### Supplementary Information

Below is the link to the electronic supplementary material.

Supplementary file1 (XLS 104 KB)

Supplementary file2 (DOCX 117 KB)

## Data Availability

These data were derived from the following public domain resources: TCGA-BRCA and the Gene Expression Omnibus (GEO) series GSE176307.
