# Supplementary material for: The 27-gene IO score is associated with efficacy of PD-1/L1 inhibitors independent of FGFR expression in a real-world metastatic urothelial carcinoma cohort
Source: Cancer Immunol Immunother. 2023 Feb 19;72(7):2075–86. doi: 10.1007/s00262-023-03401-x (PMC10264529; doi:10.1007/s00262-023-03401-x)
Supplement: Supplementary file 2 — Supplementary file2 (DOCX 117 KB) [file 262_2023_3401_MOESM2_ESM.docx]

The 27-gene IO score is associated with efficacy of PD-1/L1 inhibitors independent of FGFR expression in a real-world metastatic urothelial carcinoma cohort.

**Supplemental Data**:

Among the 108 patients with FGFR aberration UNC (n=14), we observed a clinically meaningful response for IO positivity despite the small cohort size.

| **Table S1**. TCGA-BLAD analysis with and without the gene ITM2A. | | | |
| --- | --- | --- | --- |
| Cohen’s κ = 0.99 | 26-genes IO score | | |
| 27-genes IO score | IO Score | IO+ | IO- |
|  | IO+ | 175 | 1 |
|  | IO- | 2 | 255 |

| **Figure S1**. Forest plot representing Cox proportional hazards for clinical characteristics of the cohort (n=73) from 1-year progression-free survival or 2-year overall survival. |
| --- |
|  |

| **Figure S2**. Kaplan-Meier estimates of IO score from patients with FGFR mutations or fusions. A) HR = 0.3001; 95%CI 0.08 – 1.17; p=0.082 B) HR = 0.6162; 95%CI 0.20 – 1.91; p=0.40 |
| --- |
| 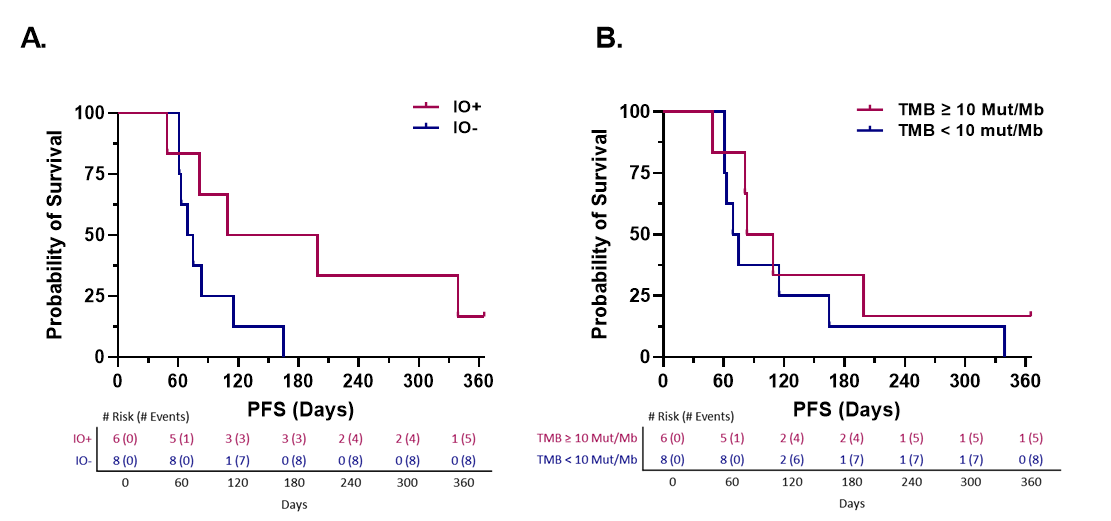 |

| **Figure S3.** 1-yr PFS ECOG 2 from UNC cohort (n=11) median PFS IO- = 52 days; IO+ = 196.5 days; HR = 0.3156; 95%CI 0.06 – 1.55; p=0.156 |
| --- |
| 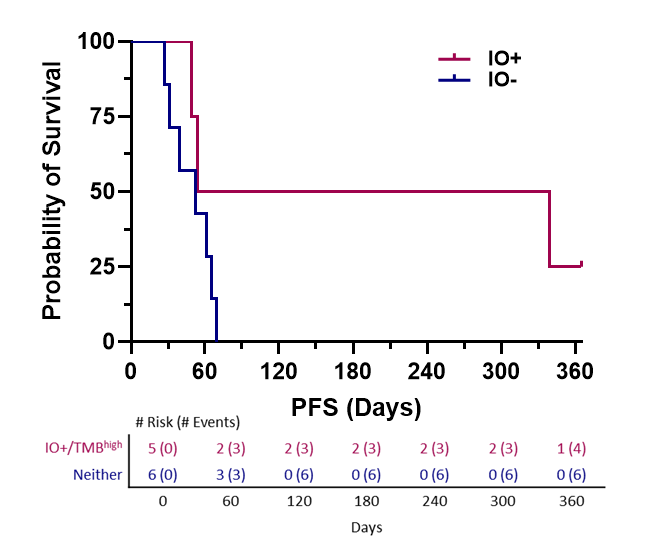 |
